# The City Coroner's Annual Report

**Published:** 1920-04-10

**Authors:** 


### THE CITY CORONER'S ANNUAL REPORT.

DURING 1910, in the City of London, including H.M. Prison at Holloway, inquests were held in the case of 158 deaths, as compared with 132 in 1918. In the borough of Southwark inquests were held in the case of 197 deaths, as compared with 177 in 1918. These figures give an excess of 46 inquests as compared with those held in 1918.

In addition to those deaths resulting in inquest, 78 investigations were made by the coroner which rendered public inquest unnecessary. *Post-mortem* examinations were ordered in 85 per cent. of inquests held. The large majority of deaths were due to accident or violence of those dying in hospitals, comparatively few deaths being the result of natural causes. Forty-four fatalities were due to vehicles, of which number 19 occurred in the City and 25 in Southwark. As suggested in former reports, the three chief preventives continue to be: (1) More street refuges, especially in Southwark; (2) more police at fixed crossings to assist children and other foot-passengers across congested streets; and (3) side life guards fixed compulsorily in all heavy commercial motors, similar to those in use on motor omnibuses.

Inquests were held on the bodies of three infants, accidentally suffocated whilst in bed with their parents, as compared with seven such deaths investigated in 1918. There has been a marked decrease throughout England and Wales during the past few years in this class of death. For instance, the numbers in 1913 were 1,176, as compared with 585 in 1917. Dr. Waldo attributes this decrease chiefly to the greater use of cots and cradles by the poor, as well as to the large and increasing number of autopsies ordered by coroners, by which means many of these deaths are proved to be natural ones. Drink, in the coroner's opinion, has little to do with these cases.

Touching the subject of anæsthetics, inquests were held in only six cases (two in the City and four in Southwark) on those whose sudden death was accelerated by the administration of anæsthetics for surgical operations. In five of these cases the drug given was chloroform, the most deadly anæsthetic known. The sixth death was due to ethyl chloride. The coroner significantly adds: "It is a noteworthy fact that in nearly every instance of this class of case coming before me during the past eighteen and a half years death has been attributed either to chloroform or to a mixture containing chloroform. These deaths, however, have, happily, considerably diminished in frequency of late years, owing largely to the greater care and skill used in the administration, and discrimination exercised in the choice, of the particular anæsthetic given."

The fires reported number thirty-three more than those of 1918. However, the number of big, serious conflagrations continues to decrease. Public inquests were held before the coroner by a jury concerning four non-fatal City fires. Dr. Waldo continues personally to investigate on the spot all the more important of the fires reported to him, which practice, he says, is of great importance in the elucidation of their origin and prevention. Thirteen of the fires investigated were due to defective electrical arrangements, and upwards of one-third of the total number of fires reported were caused by "a light thrown down."

Dr. Waldo again suggests that the City Fire Act should be extended to his jurisdiction in Southwark.

With regard to the war measure known as the Juries Act, 1918, aimed at the saving of time of business men acting as jurors, and excusing such service save in suspected cases of murder and manslaughter, deaths in prison, and in a few other cases, Dr. Waldo makes the following observations: "The war being now practically over, there is no longer a shortage of jurors, and I personally think it would be in the public interest, if the power to hold coroner's inquests without a jury be done away with, and a return made to the pre-war, uniform, constitutional, and popular method of sitting with a jury in all cases. In the City there is no lack of highly intelligent jurors, able to follow and appreciate the evidence, and ready to give valuable assistance in the administration of justice. Altogether, I am of opinion that a fairly intelligent jury, assisted and directed by a coroner, is a far better tribunal for the elucidation of truth than a coroner unassisted by a jury."

From this it would appear that the summoning of a more educated class of juror, whenever possible, is desirable, rather than the doing away with juries. The jury being members of the public enhances the value of the inquest, making it an important safeguard to life and property. The publicity of the Coroner's Court is its greatest asset, leading to the detection of guilt and acting as a deterrent of crime, whilst at the same time it tends to protect the innocent.

